# Reduction and Return of Infectious Trachoma in Severely Affected Communities in Ethiopia

**DOI:** 10.1371/journal.pntd.0000376

**Published:** 2009-02-10

**Authors:** Takele Lakew, Jenafir House, Kevin C. Hong, Elizabeth Yi, Wondu Alemayehu, Muluken Melese, Zhaoxia Zhou, Kathryn Ray, Stephanie Chin, Emmanuel Romero, Jeremy Keenan, John P. Whitcher, Bruce D. Gaynor, Thomas M. Lietman

**Affiliations:** 1 Orbis International, Addis Ababa, Ethiopia; 2 F.I. Proctor Foundation, University of California, San Francisco, United States of America; 3 Department of Ophthalmology, University of California, San Francisco, United States of America; 4 Department of Epidemiology & Biostatistics, University of California, San Francisco, United States of America; 5 Institute for Global Health, University of California, San Francisco, United States of America; Weill Medical College of Cornell University, United States of America

## Abstract

**Background:**

Antibiotics are a major tool in the WHO's trachoma control program. Even a single mass distribution reduces the prevalence of the ocular chlamydia that causes trachoma. Unfortunately, infection returns after a single treatment, at least in severely affected areas. Here, we test whether additional scheduled treatments further reduce infection, and whether infection returns after distributions are discontinued.

**Methods:**

Sixteen communities in Ethiopia were randomly selected. Ocular chlamydial infection in 1- to 5-year-old children was monitored over four biannual azithromycin distributions and for 24 months after the last treatment.

**Findings:**

The average prevalence of infection in 1- to 5-year-old children was reduced from 63.5% pre-treatment to 11.5% six months after the first distribution (*P*<0.0001). It further decreased to 2.6% six months after the fourth and final treatment (*P* = 0.0004). In the next 18 months, infection returned to 25.2%, a significant increase from six months after the last treatment (*P* = 0.008), but still far lower than baseline (*P*<0.0001). Although the prevalence of infection in any particular village fluctuated, the mean prevalence of the 16 villages steadily decreased with each treatment and steadily returned after treatments were discontinued.

**Conclusion:**

In some of the most severely affected communities ever studied, we demonstrate that repeated mass oral azithromycin distributions progressively reduce ocular chlamydial infection in a community, as long as these distributions are given frequently enough and at a high enough coverage. However, infection returns into the communities after the last treatment. Sustainable changes or complete local elimination of infection will be necessary.

**Trial Registration:**

ClinicalTrials.gov NCT00221364

## Author Summary

Trachoma is one of the leading causes of blindness in the developing world. The World Health Organization has a multi-pronged approach to controlling the ocular chlamydial infection that causes the disease, including distributing antibiotics to entire communities. Even a single community treatment dramatically reduces the prevalence of the infection. Unfortunately, infection returns back into communities after treatment, at least in severely affected areas such as rural Ethiopia. Here, we assess whether additional scheduled treatments in 16 communities in the Gurage area of Ethiopia further reduce infection, and whether the disease returns after distributions are stopped. In communities with the highest levels of trachoma ever studied, we find that repeated mass oral azithromycin distributions gradually reduce the prevalence of trachoma infection in a community, as long as these treatments are given frequently enough and to enough people in the community. Unfortunately, infection returns into the communities after the last treatment. Sustainable changes or complete local elimination of infection will be necessary to stop the return of ocular chlamydial in communities with very high prevalence of the disease.

## Introduction

Trachoma remains a leading cause of blindness in poor, arid areas such as sub-Saharan Africa[1]. The World Health Organization (WHO) has targeted the disease in a control program, relying in part on repeated mass distributions of oral azithromycin to reduce the prevalence of the ocular chlamydial infection that causes the disease[2]. The program has had remarkable success in areas with moderate levels of infection and in areas where socioeconomic improvements are occurring in concert with the trachoma program. Even a single distribution can dramatically reduce the prevalence of chlamydia[3],[4],[5],[6]. In modestly infected communities, infection may then remain low[5]. In fact, in some areas, infectious trachoma is decreasing even in the absence of treatment[7],[8],[9]. However in severely affected communities, infection clearly returns after a single mass distribution, and repeated treatments will be necessary[10],[11],[12]. It is unknown whether scheduled, repeated distributions will progressively reduce infection, and for how long distributions will need to be given[10],[13]. In a previous study in southern Ethiopia, infection returned in the first 6 months after treatment at a rate that suggested that biannual treatment would be necessary for progressive reduction with each distribution[10],[14]. Here we present longer term results from these same villages, assessing the effect of four biannual distributions, and determining whether or not infection returns after treatments have been discontinued.

## Methods

From the district record of the Enemore district of the Gurage Zone, southern Ethiopia, a simple random sample was chosen of 16 kebele (a government unit which in this area includes approximately 5 villages). A single random village was selected from each of 16 randomly chosen kebele as previously described[10]. Selected villages were given four biannual, community-wide antibiotic distributions starting in March 2003. At scheduled treatments, those aged 1 year and older were offered a single dose of directly observed, oral azithromycin (1 g in adults or 20 mg/kg in children). Pregnant women and those allergic to macrolides were offered a 6-week course of topical 1% tetracycline ointment (applied twice daily to both eyes and not directly observed).

All children aged 1–5 years in treated villages were examined and assessed for the presence of ocular chlamydial infection (defined by a positive PCR test), at baseline (pre-treatment), and 6, 12, 18, 24, 30, 36, and 42-months post-treatment. Examination was performed using the WHO's simplified grading system, with clinical activity being defined as a grade of TF (5 more follicles on the upper tarsal conjunctiva) or TI (intense inflammation of the conjunctiva obscuring more than half of the normal, underlying conjunctival vasculature)[15]. A dacron swab was passed firmly across the right upper tarsal conjunctiva three times, rotating between each pass. In a randomly selected sample of 5 children per village, a duplicate field control was taken in an identical manner to the initial swab. In a separate 5 randomly chosen children per village, a negative field control was obtained immediately after the initial swab by passing a swab within one inch of the subject's conjunctiva. Examiners then changed gloves for the next subject (whether or not the child had been selected as a control). All samples were kept at 4°C in the field and frozen at −20°C within 6 hours. The swabs were shipped at 4°C to San Francisco where they were stored at −70°C until processed. The Amplicor PCR test (Roche Diagnostics, Branchburg, NJ) was used to detect chlamydial DNA. Pre-treatment samples were tested individually. Post-treatment samples from the same village were randomized and pooled into groups of 5, with a possible remainder pool of 1–4 samples. Each pool was then tested according to the Amplicor protocol[16],[17]. If PCR of any pool was equivocal, then all samples from the pool were individually re-tested. The prevalence of ocular chlamydial infection in each village was obtained by maximum likelihood estimation[10],[12],[14]. The number of positive individual samples most likely to have resulted in the observed pooled PCR results was chosen as the estimate for that village (*Mathematica* 5.0, Wolfram Research Inc., Champaign, IL). If 2/3 or more of the pools were positive, the individual samples were re-pooled randomly into groups of two and re-processed to allow more accurate estimation[17]. Lab personnel were masked to the identification of the village and the individual. The mean village prevalence of infection at different visits was compared using the paired T-test in STATA 9.0.

**Figure 1:**
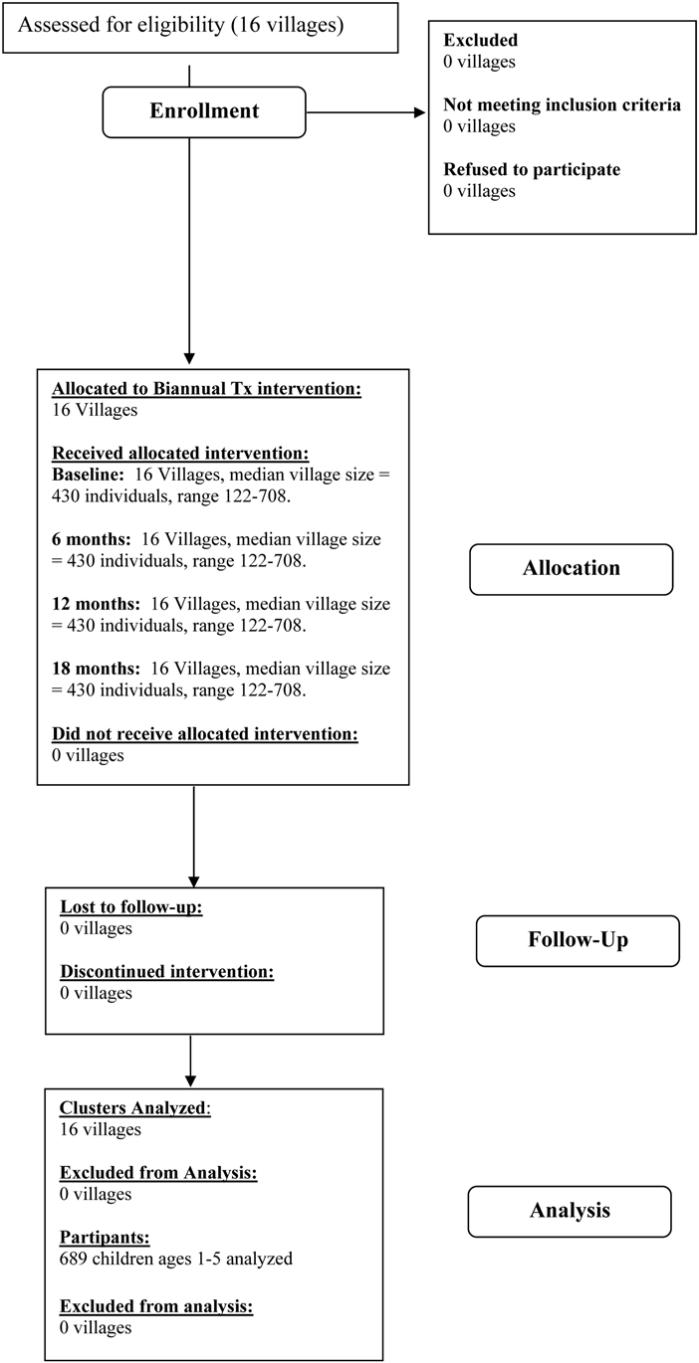
CONSORT Flowchart.

At the time of each procedure, informed consent was obtained verbally from all participants by trained Ethiopian staff in the local language, Amharic. Written consent was not deemed appropriate, given the low literacy rate in the region. Participants and guardians were assured that they would receive the same care, regardless of their participation in the monitoring program. Ethical approval for this study was obtained from the Committee for Human Research of the University of California, San Francisco and the Ethiopian Science and Technology Commission; the study was carried out in accordance with the Declaration of Helsinki. The study is registered at www.clinicaltrials.gov as trial number NCT00221364. The CONSORT protocol and checklist are noted as Figure 1 and Checklist S1, respectively.

## Results

In the baseline census, the 16 villages contained 5735 individuals in 1348 households. There were 808 1–5 year-old children, 48.6% of whom were girls (95% CI 45.3 to 51.9%). In these children, the mean prevalence of clinical activity (defined as either TF or TI in the WHO simplified grading scale), was 91.6% (95% CI 87.8 to 95.5%). The mean coverage of antibiotic at a village visit was 94.1% relative to the census, ranging from 73.9% to 100% of those eligible for treatment (Table 1). The first treatment reduced infection in all 16 villages, and the next three treatments further reduced the prevalence in 14 of 16 villages. Infection increased in the 24 months after the last treatment in 14 of 16 villages, but remained lower than it had been before the first treatment in all 16 villages. The mean prevalence was reduced from 63.5% at baseline (range 26.3% to 85.7%) to 11.5% six months after the first treatment range (0.0 to 24.4%) as previously described (*P*<0.0001)[10]. The next three biannual distributions continued to reduce the prevalence to 2.6% (range 0.0% to 7.0%), a significant decrease from after a single treatment (*P* = 0.0004). From 6 to 24 months after the last treatment (the 24 and 42 month visits), infection had returned to an average of 25.2% (range 2.0% to 70.0%). At 24 months after the last treatment, the mean prevalence of infection was far higher than at 6 months after the last treatment (*P* = 0.008), but was still only 40% of that at baseline (*P*<0.0001).

**Table 1:** Estimated Antibiotic Coverage at Each Village Visit.

Village	Total population	Antibiotic Coverage			
		Baseline (%)	6-Months (%)	12-Months (%)	18-Months (%)
**1**	523	95.8%	95.7%	98.1%	98.4%
**2**	572	84.8%	95.8%	98.0%	97.1%
**3**	365	94.5%	96.4%	94.2%	96.5%
**4**	708	95.6%	96.9%	96.6%	98.6%
**5**	621	96.7%	96.6%	93.8%	95.0%
**6**	670	95.8%	93.5%	97.4%	97.3%
**7**	386	85.9%	83.3%	83.0%	91.5%
**8**	306	97.4%	94.4%	96.2%	93.3%
**9**	241	99.6%	99.5%	100.0%	97.3%
**10**	122	97.2%	88.6%	96.8%	88.6%
**11**	213	85.6%	96.4%	88.5%	87.7%
**12**	184	73.9%	98.8%	90.8%	94.3%
**13**	183	93.0%	95.3%	99.3%	91.0%
**14**	599	85.1%	90.0%	98.0%	96.2%
**15**	290	93.4%	95.3%	100.0%	92.3%
**16**	898	96.4%	96.4%	97.1%	95.8%
**Mean**	**430**	**91.9%**	**94.6%**	**95.5%**	**94.4%**

94.1% relative to the census, ranging from 73.9% to 100%.

**Figure 2:**
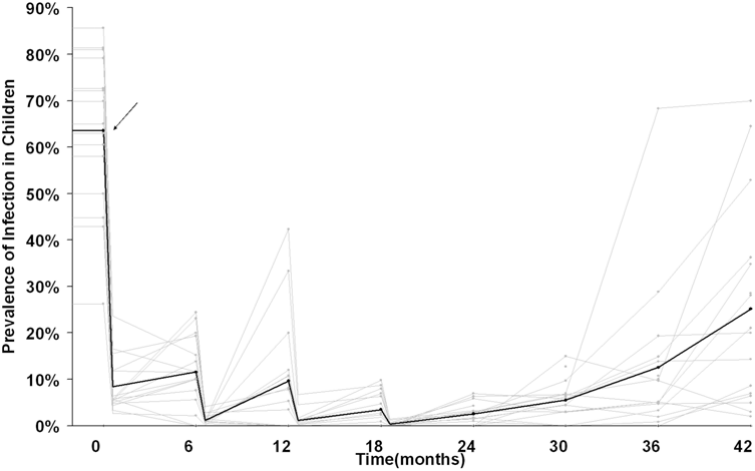
The Mean Prevalence of Infection. The mean prevalence of infection (black) and the prevalence in each of the individual 16 study villages (grey) are plotted against time. Points represent estimates of the prevalence of infection in 1–5 year-old children at a single village-visit. Lines connecting points were constructed for illustrative purposes in three ways. Before the first treatment, infection was presumed to be constant. After each treatment (black arrow), infection was presumed to decrease by the estimated coverage level, before returning in the subsequent 6 months to the observed prevalence. After the last treatment, infection was presumed to proceed linearly between each time point.

The 16 villages varied widely in their pre-treatment and post-treatment prevalence, and in how rapidly infection returned after treatments had been discontinued (Figure 2). There was a statistically significant correlation between the prevalence of infection at baseline and at 6 months (r = 0.51, *P* = 0.05), but a lower correlation between baseline and 42 months (r = 0.36, *P* = 0.17). 625 of 630 duplicate field controls were concordant (99.2%, 95% CI 98.2 to 99.7%). 625 of 632 negative field controls from baseline to the 42 month visit showed no evidence of chlamydia. (98.9%, 95% CI 97.7 to 99.6%).

## Discussion

It has been well documented that a single mass distribution of oral azithromycin to a trachoma-endemic community can lower the prevalence of ocular chlamydial infection. This has been demonstrated in group-randomized trials in Egypt, Tanzania, the Gambia, and a nearby area of Ethiopia and in observational studies in Tanzania, the Gambia, Nepal, and Vietnam[3],[4],[5],[6],[11],[12],[18]. This is true in even the most severely affected communities such as those followed in this study[10]. However, if infection is not locally eliminated, it returns back into a community after a single mass distribution, at least in severely affected areas, so repeated distributions will be necessary[10],[11],[12].

In a single, moderately affected community in Nepal, infection was reduced from 26% in children pre-treatment to a single infection (0.5%) after 3 annual treatments[4]. However, it has not been clear whether repeated, scheduled treatments can progressively reduce the prevalence of infection in the most severely affected areas. This is theoretically possible even in hyper-endemic settings, as long as distributions reduce infection more than it returns between treatments. Simple mathematical modes suggest that a progressive reduction is dependant on the coverage and frequency of distributions, and the rate that infection returns into a particular community[10],[13]. We have previously estimated that success in an area with disease as severe as seen in this part of Ethiopia could be achieved with biannual distributions [10],[13],[19]. In the 16 villages studied in this report, each subsequent treatment further lowered the mean prevalence. Infection after four treatments was significantly lower than after one treatment. Thus the prevalence of infection can be progressively reduced with each repeated distribution, at least when treatments are given biannually and at a relatively high level of coverage.

Perhaps the major unanswered question concerning mass antibiotic distributions for trachoma is whether mass antibiotic distributions can be discontinued, and if so, when[20]. If infection is not locally eliminated, will it simply return into communities after the last treatment? Some have hypothesized that infection may never return if it is brought to a low enough level, analogous to the *Allee effect* found in population biology[13],[21],[22],[23],[24]. Infection has been shown to return after a single treatment in some communities, but not in others[12],[25]. The results of this study indicate that even after four biannual treatments, if distributions are discontinued, infection predictably returns. The prevalence was lowered to an average of 2.6% prevalence after the four biannual treatments, but in the two years after the last treatment, infection had returned 10-fold to 25.2%.

There is a great deal of variation between villages, even in the same geographical area. It is tempting to speculate that underlying differences between the villages explain this variance. However, it should be noted that there is also considerable variation in the prevalence of the same village over time. Village 2 had the lowest prevalence at 12 months and one of the highest at 42 months (Table 2). Village 11 had the highest prevalence at 18 months and the lowest at 42 months. Village 9 had one of the highest prevalences pre-treatment and one of the lowest at 42 months. None of these anomalies can be explained by the treatment coverage. However, the variability of these results is well within that suggested by stochastic mathematical models, which account for the effect of chance[19],[26]. As expected in longitudinal data with unexplained variance, the correlation between each time point decreases over larger time periods. The correlation coefficient between baseline prevalence and 24 month prevalence is well below 0.5. This has important ramification for the design of trachoma studies, since it suggests that using the change of prevalence from baseline actually results in more variance (and less information) then using the post-treatment result alone[27].

**Table 2:** Estimated Prevalence of Infection at Each Village Visit.

Estimated Prevalence of Infection in 1–5 Year-Old Children %(infected/total)								
village	baseline	6 month	12 month	18 month	24 month	30 month	36 month	42 month
**1**	60.5%(23/38)	23.1%(9/39)	5.3%(2/38)	2.8%(1/36)	4.3%(2/47)	12.8%(5/39)	10.7%(6/56)	28.6%(12/42)
**2**	85.7%(60/70)	11.6%(8/69)	0.0%(0/65)	0.0%(0/62)	2.9%(1/35)	6.7%(2/30)	10.0%(3/30)	64.5%(20/31)
**3**	26.3%(10/38)	2.2%(1/45)	12.0%(6/50)	7.0%(3/43)	1.5%(1/65)	4.5%(3/67)	19.4%(13/67)	20.0%(13/65)
**4**	69.9%(65/93)	10.1%(9/89)	10.7%(11/103)	3.5%(3/86)	0.0%(0/107)	3.0%(3/100)	5.1%(6/117)	28.1%(32/114)
**5**	72.6%(45/62)	7.6%(5/66)	10.8%(7/65)	4.8%(3/63)	1.6%(1/61)	4.4%(2/45)	1.9%(1/52)	7.0%(4/57)
**6**	63.7%(58/91)	24.4%(19/78)	8.2%(9/110)	2.4%(2/84)	1.0%(1/105)	0.0%(0/118)	3.3%(4/121)	21.0%(26/124)
**7**	65.0%(39/60)	20.0%(13/65)	7.8%(4/51)	9.8%(4/41)	2.7%(2/73)	9.7%(6/62)	28.8%(21/73)	52.9%(37/70)
**8**	72.2%(26/36)	13.9%(5/36)	0.0%(0/26)	0.0%(0/34)	7.0%(3/43)	5.9%(2/34)	13.9%(5/36)	14.3%(5/35)
**9**	81.0%(17/21)	10.0%(2/20)	20.0%(6/30)	8.0%(2/25)	5.9%(2/34)	2.9%(1/34)	4.8%(2/42)	5.0%(2/40)
**10**	42.9%(6/14)	0.0%(0/13)	0.0%(0/11)	0.0%(0/18)	6.3%(1/16)	6.7%(1/15)	5.0%(1/20)	34.8%(8/23)
**11**	63.0%(17/27)	11.5%(3/26)	42.3%(11/26)	8.7%(2/23)	3.2%(1/31)	2.9%(1/34)	5.1%(2/39)	2.0%(1/51)
**12**	79.2%(19/24)	15.2%(5/33)	33.3%(8/24)	6.3%(2/32)	3.0%(1/33)	7.0%(3/43)	68.3%(28/41)	70.0%(28/40)
**13**	50.0%(8/16)	0.0%(0/13)	0.0%(0/16)	0.0%(0/16)	0.0%(0/19)	15.0%(6/40)	9.7%(3/31)	3.0%(1/33)
**14**	81.4%(57/70)	19.4%(13/67)	3.5%(2/57)	1.8%(1/56)	0.0%(0/69)	6.3%(6/95)	14.9%(13/87)	36.3%(29/80)
**15**	44.8%(13/29)	10.0%(3/30)	0.0%(0/32)	0.0%(0/43)	0.0%(0/38)	0.0%(0/55)	0.0%(0/60)	8.6%(5/58)
**16**	58.0%(69/119)	5.6%(7/126)	0.0%(0/93)	0.9%(1/108)	1.7%(2/115)	0.0%(0/113)	0.8%(1/123)	6.4%(6/94)
**mean(95%CI)**	**63.5% (54.9%–72.2%)**	**11.5% (7.5%–15.6%)**	**9.6% (2.9%–16.3%)**	**3.5% (1.6%–5.3%)**	**2.6% (1.3%–3.8%)**	**5.5% (3.2%–7.8%)**	**12.6% (3.7%–21.5%)**	**25.2% (13.5%–36.7%)**

It is not clear from where infection returns. At 21 of the 128 total village visits (16 villages×8 vists), no infection could be identified in 1–5 year-old children. At 10 of the 21 subsequent visits in the same villages, infection returned to 1–5 year-old children. Re-infection of this 1–5 year-old age group could have come from many sources. Infected individuals outside the 1–5 year-old age group would have gone undetected and could have infected 1–5 year-old children before the next treatment. Even if infection were truly locally eliminated from a community, it may be re-introduced from the outside. All communities in the district received the first two biannual distributions as part of the ORBIS trachoma program, but re-infection from neighboring areas needs to be considered. Examination of individual village results suggests that re-introduction may not occur for a period of time. We were unable to identify any infection in Village 15 for 2½ years after the second treatment. Note also that in many of the communities in this study, reinfection from neighboring villages need not even be posited, as infection clearly remained in the village itself.

The WHO-recommended mass azithromycin distributions are remarkably successful in reducing the prevalence of infectious trachoma, even in the most severely affected areas. In these 16 communities, the average prevalence of infection in 1–5 year-old children was reduced 7-fold six months after a single mass antibiotic distribution. Three subsequent biannual treatments reduced the prevalence to a total of 25-fold from baseline. Unfortunately, infection clearly returned in the 24 months after treatments were discontinued, although it had still only reached 40% of baseline prevalence. There are several ways that trachoma programs could become sustainable. Interventions such as hygiene education or latrine construction could be shown to prevent infection from returning. Although this has yet to be demonstrated, there are reasons to be optimistic, and these interventions are considered an important part of the WHO's overall trachoma strategy[28],[29],[30],[31]. Antibiotic distributions could be continued indefinitely, although this raises issues of cost, resistance, and loss of immunity[18],[32],[33],[34],[35],[36],[37]. A secular trend outside of the trachoma program may assist in the elimination of trachoma, as has been described in several other settings[7],[8],[9],[12],[25]. Finally, if infection can be eliminated locally, then this offers a higher level of sustainability[12],[19],[22].

## Supporting Information

Checklist S1 CONSORT Checklist. Statement of requirements noted within the article.
(0.11 MB DOC)
