# Supplementary material for: Reduction and Return of Infectious Trachoma in Severely Affected Communities in Ethiopia
Source: PLoS Negl Trop Dis. 2009 Feb 10;3(2):e376. doi: 10.1371/journal.pntd.0000376 (PMC2632737; doi:10.1371/journal.pntd.0000376)
Supplement: Checklist S1 — CONSORT Checklist. Statement of requirements noted within the article. (0.11 MB DOC) [file pntd.0000376.s001.doc]

# CONSORT Statement 2001 - Checklist
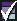


**Items to include when reporting a randomized trial**

| ***PAPER SECTION* And topic** | Item | **Descriptor** | **Reported on**  **Page #** |
| --- | --- | --- | --- |
| TITLE & ABSTRACT | 1 | [How participants were allocated to interventions](http://www.consort-statement.org/index.aspx?o=1107) (*e.g*., "random allocation", "randomized", or "randomly assigned").  Random allocation | 2 |
| *INTRODUCTION* Background | 2 | [Scientific background and explanation of rationale](http://www.consort-statement.org/index.aspx?o=1016).  Antibiotics are a major tool in the WHO’s trachoma control program. Even a single mass distribution dramatically reduces the prevalence of the ocular chlamydia that causes trachoma. Unfortunately infection returns after a single treatment, at least in severely affected areas. Here, we test whether additional scheduled treatments further reduce infection, and whether infection returns after distributions are discontinued. | 2 |
| *METHODS* Participants | 3 | [Eligibility criteria for participants](http://www.consort-statement.org/index.aspx?o=1017#3a) and the [settings and locations where the data were collected](http://www.consort-statement.org/index.aspx?o=1017#3b).  Ocular chlamydial infection in 1-5 year old children was monitored over four biannual azithromycin distributions and for 24 months after the last treatment.  From the district record of the Enemore district of the Gurage Zone, southern Ethiopia, a simple random sample was chosen of 16 kebele (a government unit which in this area includes approximately 5 villages). A single random village was selected from each of 16 randomly chosen kebele as previously described[10]. | 2, 3 |
| Interventions | 4 | [Precise details of the interventions intended for each group and how and when they were actually administered](http://www.consort-statement.org/index.aspx?o=1021).  [The sixteen] selected villages were given four biannual, community-wide antibiotic distributions starting in March 2003. At scheduled treatments, those aged 1 year and older were offered a single dose of directly observed, oral azithromycin (1g in adults or 20mg/kg in children). Pregnant women and those allergic to macrolides were offered a 6-week course of topical 1% tetracycline ointment (applied twice daily to both eyes and not directly observed). | 3-4 |
| Objectives | 5 | [Specific objectives and hypotheses](http://www.consort-statement.org/index.aspx?o=1022).  Here we present longer term results from these same villages, assessing the effect of four biannual distributions, and determining whether or not infection returns after treatments have been discontinued. | 3 |
| Outcomes | 6 | [Clearly defined primary and secondary outcome measures](http://www.consort-statement.org/index.aspx?o=1023#6a) and, when applicable, any [methods used to enhance the quality of measurements](http://www.consort-statement.org/index.aspx?o=1023#6b) (*e.g.*, multiple observations, training of assessors).  In a randomly selected 5 children per village, a duplicate field control was taken in an identical manner to the initial swab. In a separate 5 randomly chosen children per village, a negative field control was obtained immediately after the initial swab by passing a swab within one inch of the subject's conjunctiva. | 4 |
| Sample size | 7 | [How sample size was determined](http://www.consort-statement.org/index.aspx?o=1024#7a) and, when applicable, [explanation of any interim analyses and stopping rules](http://www.consort-statement.org/index.aspx?o=1024#7b).  We estimated that with 16 villages, we would have 80% power to detect a 4% difference between two visits, based on previous estimates of the variance between communities and the correlation between two visits in the same village (two-tailed alpha=0.05). | - |
| Randomization -- Sequence generation | 8 | [Method used to generate the random allocation sequence, including details of any restrictions](http://www.consort-statement.org/index.aspx?o=1025) (*e.g*., blocking, stratification)  Stratified Random Sample | 5 |
| Randomization -- Allocation concealment | 9 | [Method used to implement the random allocation sequence](http://www.consort-statement.org/index.aspx?o=1026) (*e.g*., numbered containers or central telephone), clarifying whether the sequence was concealed until interventions were assigned.  RANDOM() in Microsoft Excel | - |
| Randomization -- Implementation | 10 | [Who generated the allocation sequence, who enrolled participants, and who assigned participants to their groups](http://www.consort-statement.org/index.aspx?o=1027).  Generated by TML, enrolled and assigned by MM | - |
| Blinding (masking) | 11 | [Whether or not participants, those administering the interventions, and those assessing the outcomes were blinded to group assignment](http://www.consort-statement.org/index.aspx?o=1028#11a). If done, [how the success of blinding was evaluated](http://www.consort-statement.org/index.aspx?o=1028#11b).  Lab personnel were masked to the identification of the village and the individual. | 5 |
| Statistical methods | 12 | [Statistical methods used to compare groups for primary outcome(s)](http://www.consort-statement.org/index.aspx?o=1029#12a); [Methods for additional analyses](http://www.consort-statement.org/index.aspx?o=1029#12b), such as subgroup analyses and adjusted analyses.  The next three biannual distributions continued to reduce the prevalence to 2.6% (range 0.0% to 7.0%), a significant decrease from after a single treatment (paired T-test, P=0.0004). | 5 |
| RESULTS Participant flow | 13 | [Flow of participants through each stage](http://www.consort-statement.org/index.aspx?o=1018) (a diagram is strongly recommended). Specifically, for each group report the numbers of participants randomly assigned, receiving intended treatment, completing the study protocol, and analyzed for the primary outcome. [Describe protocol deviations from study as planned, together with reasons](http://www.consort-statement.org/index.aspx?o=1086).  We explained in the text itself, since the protocol had only a single arm:  [In the 16 villages] there were 808 1-5 year-old children, 48.6% of whom were girls (95% CI 45.3 to 51.9%). The mean coverage of antibiotic at a village visit was 94.1% relative to the census, ranging from 73.9% to 100% of those eligible for treatment (Table 1). | 5 |
| Recruitment | 14 | [Dates defining the periods of recruitment and follow-up](http://www.consort-statement.org/index.aspx?o=1087).  Selected villages were given four biannual, community-wide antibiotic distributions starting in March 2003 (and lasting 42 months). | 3-4 |
| Baseline data | 15 | [Baseline demographic and clinical characteristics of each group](http://www.consort-statement.org/index.aspx?o=1088).  In the baseline census, the 16 villages contained 5735 individuals in 1348 households. There were 808 1-5 year-old children, 48.6% of whom were girls (95% CI 45.3 to 51.9%). | 5 |
| Numbers analyzed | 16 | [Number of participants (denominator) in each group included in each analysis and whether the analysis was by "intention-to-treat"](http://www.consort-statement.org/index.aspx?o=1089). State the results in absolute numbers when feasible (*e.g*., 10/20, not 50%).  In the baseline census, the 16 villages contained 5735 individuals in 1348 households. There were 808 1-5 year-old children, 48.6% of whom were girls (95% CI 45.3 to 51.9%). | 5 |
| Outcomes and  estimation | 17 | [For each primary and secondary outcome, a summary of results for each group, and the estimated effect size and its precision](http://www.consort-statement.org/index.aspx?o=1090) (*e.g.*, 95% confidence interval).  The results for all 16 villages were expressed in Table and Graph format. The P-value for comparisons between visits and the correlation coefficient between a village’s prevalence at two different visits were reported. | 6-7,  Table, and  Figure |
| Ancillary analyses | 18 | [Address multiplicity by reporting any other analyses performed](http://www.consort-statement.org/index.aspx?o=1091), including subgroup analyses and adjusted analyses, indicating those pre-specified and those exploratory.  We reported 6 P-values, and calculated 2 additional P-values that have not been reported. The first 4 reported P-values were comparisons between the mean prevalence of infection at different visits, and would remain significant even with a strict, Bonferroni correction. The correlation between the prevalence of infection at baseline and at 6-months (*r*=0.51, *P*=0.05) is more vulnerable to a multiple comparisons correction, but our point is simply that this correlation decreases with time, to (*r*=0.36, *P*=0.17) at 42 months. | 5-6 |
| Adverse events | 19 | [All important adverse events or side effects in each intervention group](http://www.consort-statement.org/index.aspx?o=1092). | - |
| *DISCUSSION* Interpretation | 20 | [Interpretation of the results](http://www.consort-statement.org/index.aspx?o=1019), taking into account study hypotheses, sources of potential bias or imprecision and the dangers associated with multiplicity of analyses and outcomes.  Discussion of the variance in outcome on pages 7-8. Discussion of source of recurrent infection, page 8. | 7-8 |
| Generalizability | 21 | [Generalizability (external validity) of the trial findings](http://www.consort-statement.org/index.aspx?o=1094).  In discussion: There are several ways that trachoma programs could become sustainable. Interventions such as hygiene education or latrine construction could be shown to prevent infection from returning. Although this has yet to be demonstrated, there are reasons to be optimistic, and these interventions are considered an important part of the WHO’s overall trachoma strategy[24-27]. Antibiotic distributions could be continued indefinitely, although this raises issues of cost, resistance, and loss of immunity[17, 28-33]. A secular trend outside of the trachoma program may assist in the elimination of trachoma, as has been described in several other settings[3, 4, 8, 9, 12]. Finally, if infection can be eliminated locally, then this offers a higher level of sustainability[12, 18, 20]. | 8-9 |
| Overall evidence | 22 | [General interpretation of the results in the context of current evidence](http://www.consort-statement.org/index.aspx?o=1095).  In discussion. The WHO-recommended mass azithromycin distributions are remarkably successful, even in the most severely affected areas. In these 16 communities, the average prevalence of infection in pre-school children was reduced 7-fold six months after a single mass antibiotic distribution. Three subsequent biannual treatments reduced it a total of 25-fold from baseline. Unfortunately, infection clearly returned in the 24 months after treatments were discontinued, although it had still only reached 40% of baseline prevalence. | 8-9 |
|  |  |  |  |

**www.consort-statement.org**
